# Single-cell RNA-seq of primary bone marrow neutrophils from female and male adult mice

**DOI:** 10.1038/s41597-022-01544-7

**Published:** 2022-07-23

**Authors:** Minhoo Kim, Ryan J. Lu, Bérénice A. Benayoun

**Affiliations:** 1 Leonard Davis School of Gerontology, University of Southern California, Los Angeles, CA USA; 2 Graduate Program in the Biology of Aging, University of Southern California, Los Angeles, CA USA; 3 Molecular and Computational Biology Department, USC Dornsife College of Letters, Arts and Sciences, Los Angeles, CA USA; 4 Biochemistry and Molecular Medicine Department, USC Keck School of Medicine, Los Angeles, CA 90089 USA; 5 USC Norris Comprehensive Cancer Center, Epigenetics and Gene Regulation, Los Angeles, CA USA; 6 USC Stem Cell Initiative, Los Angeles, CA USA

**Keywords:** Neutrophils, Gene expression, Immunogenetics

## Abstract

Widespread sex-dimorphism is observed in the mammalian immune system. Consistently, studies have reported sex differences in the transcriptome of immune cells at the bulk level, including neutrophils. Neutrophils are the most abundant cell type in human blood, and they are key components of the innate immune system as they form a first line of defense against pathogens. Neutrophils are produced in the bone marrow, and differentiation and maturation produce distinct neutrophil subpopulations. Thus, single-cell resolution studies are crucial to decipher the biological significance of neutrophil heterogeneity. However, since neutrophils are very RNA-poor, single-cell profiling of these cells has been technically challenging. Here, we generated a single-cell RNA-seq dataset of primary neutrophils from adult female and male mouse bone marrow. After stringent quality control, we found that previously characterized neutrophil subpopulations can be detected in both sexes. Additionally, we confirmed that canonical sex-linked markers are differentially expressed between female and male cells across neutrophil subpopulations. This dataset provides a groundwork for comparative studies on the lifelong transcriptional sexual dimorphism of neutrophils.

## Abstract (summary)

**Table:** 

Measurement(s)	RNA-seq of coding RNA from single cells
Technology Type(s)	RNA sequencing
Factor Type(s)	Genotype
Sample Characteristic - Organism	Mus musculus
Sample Characteristic - Environment	laboratory facility

## Background & Summary

The mammalian immune system displays widespread sex dimorphism^1–3^. In general, males are more susceptible to and have worse outcomes for severe infections, whereas females are more prone to autoimmune diseases^3–6^. Consistently, transcriptome profiling studies have reported strong sex differences in the gene expression patterns of immune cells throughout life, including neutrophils^7,8^. Neutrophils are key elements of the innate immune system that constitute the first line of defense in response to inflammatory stimuli^9^. Neutrophils protect the host by phagocytosis, production of antimicrobial granules, and release of neutrophil extracellular traps^10–12^. Consequently, neutrophil dysfunction has been linked to the pathogenesis of various diseases, including atherosclerosis, macular degeneration, and cancer^13–15^. Recently, our group performed a multi-omic study, including bulk RNA-seq analysis, of murine bone marrow neutrophils with respect to sex and age, and we observed significant sex differences in the neutrophil transcriptome and functional landscape throughout life^8^. Additionally, bulk RNA-seq analysis of murine spleen neutrophils by the ImmGen Consortium revealed transcriptional differences between female and male animals, and such sex differences were enhanced upon interferon stimulation^7^. Together, these findings suggest that neutrophils harbor sex dimorphic gene regulation.

Neutrophils are constantly produced in the bone marrow, and they migrate to the infected site through the circulatory system^16,17^. Differentiation and maturation of neutrophils have been shown to produce distinct neutrophil subpopulations^16,18,19^. Consequently, single-cell resolution approaches are essential to explore neutrophil heterogeneity and understand neutrophil biology. However, due to the low RNA and high RNAse content of neutrophils, special precautions need to be taken to capture and robustly profile neutrophils in single-cell RNA-seq protocols^20,21^. A recent landmark study by Xie *et al*. performed single-cell transcriptome profiling of neutrophils purified by flow cytometry from the bone marrow, peripheral blood and spleen of *female* mice^22^. The study identified eight distinct neutrophil subpopulations (G0-G4 and G5a-c) at different stages of maturation in homeostatic state and proportions of each neutrophil subpopulation were different among bone marrow, peripheral blood and spleen^22^. In the bone marrow, relatively immature neutrophil subpopulations (G0-G4) were detected as more abundant, whereas more mature neutrophils (G5a-c) were found at higher frequency in the peripheral blood and spleen^22^. Additionally, Xie *et al*. leveraged the single-cell RNA-seq dataset to capture dynamic transitions between neutrophil subpopulations upon bacterial infection^22^. To gain insights on the regulatory networks and potential contribution of distinct neutrophil subpopulations to the observed sex differences in the neutrophil transcriptional landscape, single-cell resolution approaches are necessary. However, single-cell resolution datasets of neutrophil transcriptional landscapes that include both sexes are still lacking.

**Fig. 1:**
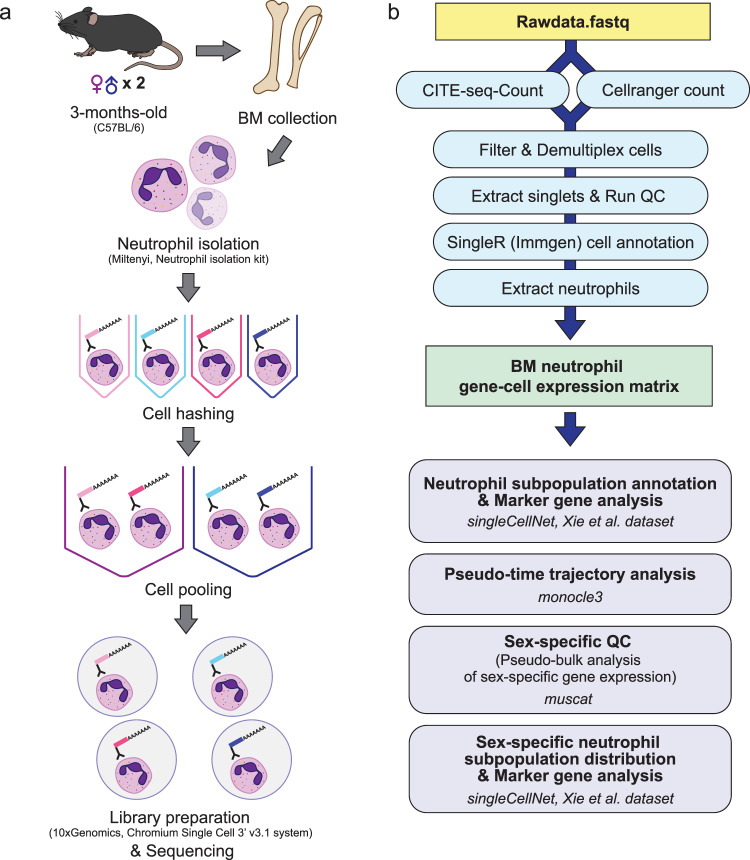
Outline of sample preparation and data analysis workflow. (**a**) Outline of sample preparation workflow. Bone marrow was collected from two female and two male 3-month-old C57BL/6 mice. Isolated neutrophils were labelled with HTOs and pooled for sequencing library preparation and sequencing. (**b**) Outline of data analysis workflow. HTO counts and gene-barcode matrices were quantified using CITE-seq-Count^31^ and Cellranger count^29^ functions, respectively. After demultiplexing, singlet neutrophils (annotated using SingleR^32^ and ImmGen^33^ database) with gene count greater than 100 and mitochondrial gene count less than 25% were extracted to obtain a clean gene-cell expression matrix. Neutrophil subpopulation annotation and marker gene analyses were performed using singleCellNet^39^ and the Xie *et al*. dataset^34^. Sex-specific gene expression was quantified via pseudo-bulk analysis using muscat^41^. Pseudo-time trajectory analysis was performed using monocle3^40^. HTO: Hash tag oligo. QC: Quality control.

Here, we generated a single-cell RNA-seq dataset of primary neutrophils from 3-month-old female and male adult mouse bone marrow with biological replicates (n = 2 per sex) (Fig. 1a and Table 1). All the samples in our dataset were handled and processed together to eliminate potential batch effects, including the use of cell hashing^23^. Single-cell multiplexing provides multiple benefits, including, but not limited to, mitigating batch effects and reducing costs^23^. Through technical validation, as described below, we demonstrate technical quality of our dataset. After quality control, we annotated the neutrophil subpopulations by utilizing the annotation scheme from the study by Xie *et al.*^22^ - we identified comparable proportions of neutrophil subpopulations in our bone marrow neutrophil dataset as in the bone marrow neutrophil data from the original study. Additionally, we performed differential expression analysis of key marker genes for each neutrophil subpopulation between female and male bone marrow neutrophils. Overall, our dataset represents an important resource to investigate sex dimorphism in neutrophil biology.

**Table 1:** Sample information.

Sample	Sex	Age	HTO sequence
3m_F_1	Female	3-months-old	HTO1-ACCCACCAGTAAGAC
3m_M_1	Male		HTO2-GGTCGAGAGCATTCA
3m_F_2	Female		HTO3-CTTGCCGCATGTCAT
3m_M_2	Male		HTO4-AAAGCATTCTTCACG

## Methods

### Mouse husbandry

All animals were treated and housed in accordance with the Guide for Care and Use of Laboratory Animals. All experimental procedures were approved by the University of Southern California (USC)’s Institutional Animal Care and Use Committee (IACUC) and are in accordance with the institutional and national guidelines. Samples were derived from animals on approved IACUC protocol #20804. Adult female and male C57BL/6 J mice (3-month-old animals) were obtained from Jackson Laboratories. Animals were acclimated at the animal facility at USC for 2 weeks before euthanasia. The animal facility at USC is on a 12-h light/dark cycle and animal housing rooms are maintained at 22 °C and 30% humidity. All animals were euthanized by CO_2_ asphyxiation followed by cervical dislocation.

### Isolation of primary neutrophils from the mouse bone marrow

Mouse bone marrow neutrophils were isolated using a previously described protocol that yields highly pure neutrophils^8,24^. Specifically, hind limb long bones of each mouse were collected and kept on ice in D-PBS (Corning) supplemented with 1% antibiotic-antimycotic (Gibco) until further processing. Muscle and connective tissues were removed from the bones, and the bone marrow from the cleaned bones was collected into clean tubes by centrifugation^25^. Red blood cells from the bone marrow were removed using the Red Blood Cell Lysis buffer (Miltenyi Biotec, no. 130-094-183), according to the manufacturer’s instructions, albeit with no vortexing to avoid arbitrary neutrophil activation. The suspension was filtered on 70-μm mesh filters (Miltenyi Biotec, no. 130-110-916) to retain only single cells for downstream processing. Neutrophils were isolated from other bone marrow cells using MACS (Miltenyi Biotec, no. 130-097-658). Viability and yield were assessed using trypan blue exclusion and an automated COUNTESS cell counter (Thermo Fisher Scientific). Purified cells were immediately used for cell hashing.

### Cell hashing, single-cell RNA-seq library preparation & sequencing

Cell hashing was performed using TotalSeq^TM^-A Antibodies (BioLegend) according to the manufacturer’s instructions (TotalSeq^TM^-A Antibodies and Cell Hashing with 10x Single Cell 3′ Reagent Kit v3 or v3.1 (Single Index) Protocol) (Table 1). Specifically, 1 million purified neutrophils were first incubated with mouse Fc Blocking Reagent (Miltenyi Biotec, no. 130-092-575) at 4 °C for 10 minutes. Then, each blocked sample was incubated with 1 μg of specific TotalSeq Cell Hashing antibody (HTOs 1-4) at 4 °C for 30 minutes. After incubation, stained cells were washed 3 times, and assessed for viability and yield using the COUNTESS cell counter (Thermo Fisher Scientific). Female neutrophil samples (HTO 1, 3) and male neutrophil samples (HTO 2, 4) were pooled separately by sex of origin in equal volumes.

Single-cell RNA-seq libraries were generated using the Chromium Next GEM Single-cell 3′ v3.1 assay (10xGenomics) according to the manufacturer’s instructions (10xGenomics User Guide Chromium Next GEM Single-cell 3′ Reagent Kits v3.1 (CG000204, Rev D))^26^ with modifications to accommodate for low RNA content of neutrophils. Briefly, cell numbers equivalent to a target cell recovery of 5,000 cells per sample after sequencing were loaded onto a 10x Genomics Chromium Next GEM G Chip with the reverse transcription enzyme master mix. We used the Chromium Next GEM Single-cell 3′ Reagent Kits v3.1 to perform mRNA capture, barcoding and reverse transcription within the GEMs. Single-cell RNA-seq libraries were generated using the Single-cell 3′ Reagent Kit. To allow for library complexity despite low input RNA, an additional 2 cycles were added to the cDNA amplification step (for a total of 14 cycles). HTO Additive primer v2 (Integrated DNA Technologies) was added to the cDNA amplification reaction to amplify the HTO molecules. The amplified HTOs were recovered in the cleanup step for the cDNA amplification, and HTO libraries were built using the 2x NEBNext PCR Master Mix and Truseq indexing oligos (Integrated DNA Technologies).

Single-cell RNA-seq and HTO libraries were quantified and quality controlled using a Qubit fluorometer and the 4200 TapeStation system (Agilent Technologies) with a High Sensitivity DNA ScreenTape. Paired-end reads (26 + 8 + 98 bp) were generated on the Illumina NextSeq550 platform at the USC Genome Core.

### Bioinformatic analysis

For sequencing data analysis, we followed the best-practice recommendations for single-cell RNA-seq analysis (Fig. 1b). Our pipeline was tested and validated in R^27^ versions 3.6.3 and 4.1.2 and Seurat^28^ versions 3.2.2 and 4.1.0. We report here the results using R 4.1.2 and Seurat 4.1.0. Each step of our bioinformatic analysis pipeline is discussed in detail below.

#### Filter and demultiplex cells

Raw reads were aligned to the mouse genome (mm10) and processed using the “cellranger count” (6.0.2) pipeline^29^ (--*expect-cells* = *5000* --*include-introns*) to generate gene-barcode matrices (Tables 2 and 3). We used the --*include-introns* option to account for the limited number of genes expressed in neutrophils^30^, as recommended by 10xGenomics. HTO quantification was performed using CITE-seq-Count (v. 1.4.5)^31^ with the following parameters: *-cbf 1 -cbl 16 -umif 17 -umil 26 -cells 5000* (Table 4). Gene-barcode matrices and HTO counts were loaded into R and cells that were detected by both RNA and HTO were filtered for downstream analysis. Additionally, genes that were not detected in at least 20 cells were excluded to eliminate possible random noise. After setting up a Seurat^28^ object using the gene-barcode matrices, HTO counts were added to the Seurat object as an independent assay and normalized using the centered log-ratio (CLR) transformation. Cells were demultiplexed using the MULTIseqDemux() function into four samples according to their HTO barcode of origin.

**Table 2:** Detailed QC report of 10x Genomics sequencing files (Cell Ranger).

Sample	Reads	Q30 Bases in Barcode	Q30 Bases in RNA Read	Q30 Bases in UMI	Confident Mapping to Genome	Confident Mapping to Transcriptome
Female Pool	199,687,860	96.9%	90.6%	95.8%	94.7%	87.3%
Male Pool	215,735,119	97.1%	90.9%	96.0%	95.0%	88.0%

**Table 3:** Sequencing statistics of 10x Genomics libraries (Cell Ranger).

Sample	Estimated Number of Cells	Mean Reads per Cell	Median Genes per Cell	Median UMI per Cell	Fraction Reads in Cells	Sequencing Saturation
Female Pool	2,848	70,115	1,691	5,712	96.8%	87.9%
Male Pool	3,630	59,431	1,544	4,755	97.3%	87.7%

**Table 4:** QC and information for HTO libraries.

Sample	Reads	Mean Quality Score	Matched Hashtags
Female HTO	1,722,815	27.2	79%
Male HTO	2,042,585	27.4	82%

#### Extract singlets and run QC

Singlets were retained for downstream analyses based on the MULTIseqDemux() annotations. For quality control, cells that have unique feature counts less than 100 or mitochondrial gene count greater than 25% were excluded (*subset* = *nFeature_RNA* > *100 & percent.mito* < *25*) (Table 5). After removing the unwanted cells, the dataset was normalized using the NormalizeData() function with the following parameters: *normalization.method* = *“LogNormalize”, scale.factor* = 10000.

**Table 5:** Final per sample information after HTO demultiplexing, singlet and neutrophil filtering.

Sample	QC Neutrophils	Median Genes per Neutrophil	Median UMI per Neutrophil	Median HTO per Neutrophil	Mean Mitochondrial Reads
3m_F_1	1,396	1,662	5,552	331	<0.5%
3m_F_2	1,205	1,737	5,980	208	<0.5%
3m_M_1	1,802	1,541	4,716	273	<0.5%
3m_M_2	1,622	1,540	4,765	260	<0.5%

#### Dimensional reduction and clustering

Prior to dimensional reduction, the dataset was scaled using the SCTransform() function with *vars.to.regress* = *c(“nFeature_RNA”, “percent.mito”)*. The first 15 principal components (PCs) were used for Uniform Manifold Approximation and Projection (UMAP) (RunUMAP()) and clustering (FindNeighbors() and FindClusters(), *resolution* = *0.3*).

#### SingleR cell annotation

To eliminate possible contaminants (non-neutrophil cells) from the dataset, quality controlled singlets were annotated using SingleR v.1.8.1^32^. The ImmGen expression dataset from celldex was used as the reference for cell annotation^33^. 6,025 cells out of 6,073 cells from the dataset were annotated as neutrophils (~99.21% purity). Cells annotated as non-neutrophil cells were excluded for downstream analysis.

#### Neutrophil subpopulation annotation and marker gene analysis

Neutrophil heterogeneity has been reported by multiple studies^16,18,19^. A recent work by Xie *et al*. characterized neutrophil subpopulations (G0-G4 and G5a-c) in the murine bone marrow via single-cell RNA-seq analysis of flow cytometry^22^ (hereafter referred to as the “Xie *et al*. dataset”^34^). To annotate the neutrophil subpopulations in our dataset, we leveraged the gene-barcode matrix (GSE137539^34^) and cell annotation data (GSM4081545^35^, GSM4081546^36^, GSM4081547^37^ and GSM4081548^38^) from the Xie *et al*. dataset provided in the Gene Expression Omnibus (GEO) database. singleCellNet v.0.1.0 was used to classify neutrophil subpopulations within our dataset^39^. After extracting the common genes found in both our dataset and the Xie *et al*. dataset, we trained a classifier using the scn_train() function with the following parameters: *nTopGenes* = *100, nTopGenePairs* = *50, nRand* = *50, nTrees* = 1000. scn_predict() and get_cate() functions were used to classify cells within our dataset and assign annotation to each cell, respectively. Marker genes for neutrophil subpopulations identified from the Xie *et al*. dataset (17 genes) were used to analyze marker gene expression in our dataset^22^. Dot plots of marker gene expression levels were generated using the DotPlot() function from Seurat^28^.

#### Pseudo-time trajectory analysis

Single-cell trajectory was constructed using monocle3 v.1.0.0^40^. After learning the principal graph (learn_graph()), cells were ordered using order_cells (*root_cells* = *“G2”*). “G2” subpopulation was used as root cells as they were the least matured neutrophil subpopulation detected in our dataset^22^.

#### Pseudo-bulk analysis of sex-specific gene expression

As a quality control, we assessed the expression of sex-specific genes, *Xist* and *Ddx3y*, in each neutrophil subpopulation using pseudo-bulk analysis with muscat v.1.5.2^41^. Each neutrophil subpopulation single-cell data was aggregated to pseudo-bulk data using the aggregateData() function with the *fun* = *“sum”* option. Differential state was assessed using the pbDS() function with the following parameters: *method* = *“edgeR”, min_cells* = 8 (edgeR v. 3.36.0).

#### Dimensional reduction of pseudo-bulk analysis data

To assess the potential global sex differences in the transcriptional landscapes between female and male neutrophils (globally and for each subpopulation), muscat v.1.5.2^41^ was used to perform Multidimensional Scaling (MDS, pbMDS()) of the aggregated pseudo-bulk data (output of aggregateData(), described above).

## Data Records

Sequencing data have been submitted to the Sequence Read Archive accessible through BioProject PRJNA796634^42^ (BioSamples SAMN24905300^43^, SAMN24905301^44^, SAMN24905302^45^ and SAMN24905303^46^; Table 6). We used publicly available neutrophil single-cell RNA-seq annotation data from the GEO (GSE137539^34^, 8-to-10-week-old female mouse bone marrow neutrophil samples) to annotate neutrophil subpopulations within our dataset. The final annotated Seurat object^47^ has been made available on Figshare for use and analysis (10.6084/m9.figshare.19623978).

**Table 6:** Raw sequencing data accession.

Library	BioProject	BioSample
Female Neutrophils	PRJNA796634^42^	SAMN24905300^43^
Male Neutrophils	PRJNA796634^42^	SAMN24905301^44^
Female HTO	PRJNA796634^42^	SAMN24905302^45^
Male HTO	PRJNA796634^42^	SAMN24905303^46^

## Technical Validation

### Quality control of the single-cell RNA-seq dataset

**Fig. 2:**
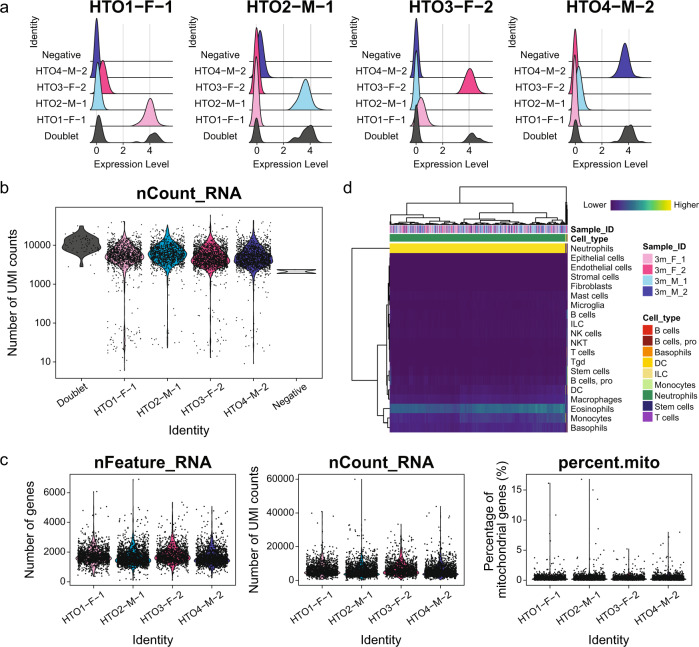
Single-cell RNA-seq dataset quality assessment. (**a**) Ridgeplots of HTO sample enrichment after demultiplexing. (**b**) Violin plot of UMI counts (nCount_RNA) from each sample after demultiplexing. (**c**) Violin plots of gene counts (nFeature_RNA, left panel), UMI counts (nCount_RNA, middle panel) and percentage of mitochondrial gene counts (pecent.mito, right panel) after quality control filtering. (**d**) Heatmap of cell annotation scores and cell annotation via SingleR^32^ and ImmGen^33^ database. HTO: Hash tag oligo. UMI: Unique molecular identifier.

**Fig. 3:**
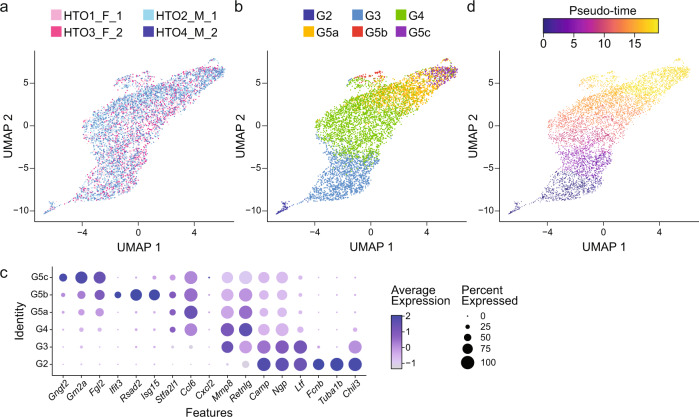
Neutrophil subpopulation annotation and marker gene analysis. (**a**), (**b**) and (**d**) Two-dimensional cell clustering via UMAP using the first 15 principal components. Cells are labelled by (**a**) HTO labels, (**b**) neutrophil subpopulations and (**d**) monocle3^40^ pseudo-time trajectory scores. (**c**) Dot plot of scaled expression levels of neutrophil subpopulation marker genes derived from the Xie *et al*. dataset^34^. UMAP: Uniform Manifold Approximation and Projection.

We utilized cell hashing^23^ to multiplex our neutrophil single-cell RNA-seq samples. Biological replicates (n = 2 animals per sex) of each sex were pooled separately for sequencing library preparation (Fig. 1a). Hash tag oligo (HTO) quantification was performed using CITE-seq-Count^31^ (Fig. 1b). As shown in Fig. 2a, we confirmed clear enrichment of each HTO (HTO1, HTO2, HTO3 and HTO4) after demultiplexing using the MULTIseqDemux() function from Seurat^28^, indicating successful cell hashing of neutrophils. Additionally, comparable Unique Molecular Identifier (UMI) counts were observed among four samples prior to quality control (Fig. 2b). To exclude potential low-quality data and noise, doublets and negative cells and cells with gene count less than 100 or mitochondrial gene count greater than 25% were excluded from the dataset (Fig. 2c). Additionally, to eliminate non-neutrophil cells from the dataset, we used SingleR^32^ and the ImmGen^33^ database to annotate the cells (Fig. 2d). Cells annotated as neutrophils (6,025 cells out of 6,073 cells from the post-filter dataset; ~99.21%) were extracted for downstream analysis (Table 5). We used the filtered neutrophil singlets to perform dimensional reduction via Uniform Manifold Approximation and Projection (UMAP) using the first 15 principal components (Fig. 3a). We did not find any noticeable sample origin-related clustering and observed a homogeneous distribution of cells derived from female and male animals (Fig. 3a).

### Annotation of neutrophil subpopulations

Recently, Xie *et al*. identified eight distinct neutrophil subpopulations (G0-4 and G5a-c) through a single-cell RNA-seq analysis of the bone marrow, peripheral blood, and spleen neutrophils from female mice^22^. In the same study, each subpopulation was identified as the following neutrophil subsets in different maturation states: G0 – granulocyte-monocyte progenitors, G1 – committed neutrophil progenitors, G2 – pre-neutrophils, G3 – immature neutrophils and G4, G5a-c – mature neutrophils^22^. To identify and annotate neutrophil subpopulations in our dataset, we used singleCellNet^39^ and the annotation information from the Xie *et al*. dataset. As shown in Fig. 3b, we identified six neutrophil subpopulations, G2-4 and G5a-c, within our dataset. Importantly, G0 and G1 subpopulations, granulocyte-monocyte progenitors and committed neutrophil progenitors, respectively, were not detected in our dataset, most likely due to depletion of progenitors by negative selection during the MACS neutrophil isolation step of our workflow^8^. To note, G0 and G1 subpopulations were detected only in small proportions in the bone marrow data of the Xie *et al*. dataset^22^. We also assessed the expression levels of marker genes associated with each neutrophil subpopulation. In the Xie *et al*. dataset, 18 marker genes were identified for subpopulations G2-4 and G5a-c. We observed similar relative expression levels of the marker genes among the neutrophil subpopulations in our dataset as was shown for the Xie *et al*. dataset, except for *Gm5483*, which was not detected in our dataset, and the expression of marker genes with respect to each neutrophil subpopulation was not grossly affected by the biological sex (Fig. 3c and Supplementary Figure 1a). Additionally, we used monocle3^40^ to construct a single-cell trajectory along pseudo-time to validate the temporal relationships among the neutrophil subpopulations along the maturation process. As shown in Fig. 3d, we confirmed neutrophil maturation along the constructed trajectory in pseudo-time, starting from G2 through G5a-c, as was described for the Xie *et al*. dataset^22^.

### Expression analysis of sex-linked genes

**Fig. 4:**
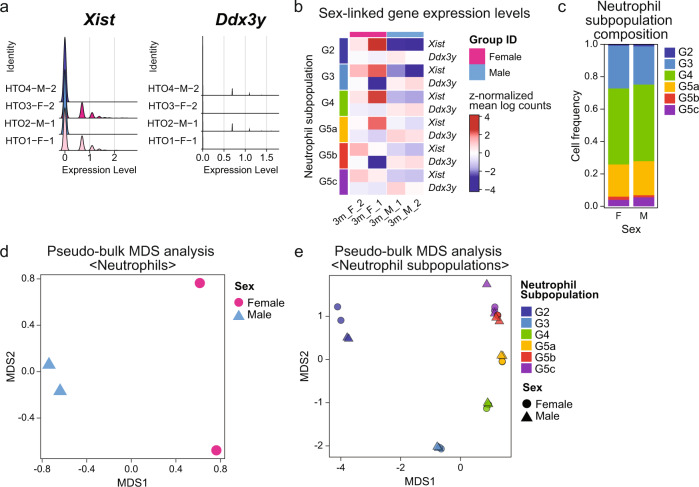
Sex-specific gene expression and neutrophil subpopulation distribution analysis. (**a**) Ridge plots of expression levels of *Xist* (female-specific) and *Ddx3y* (male-specific). (**b**) Heatmap of expression levels of sex-specific genes in each neutrophil subpopulations quantified by muscat^41^ pseudo-bulk analysis. (**c**) Percent stacked barplot of neutrophil subpopulation distribution in female and male samples. (**d**) and (**e**) MDS plots of female vs. male neutrophil pseudo-bulk data for all neutrophils (**d**) and separated by neutrophil subpopulations (**e**). F: Female. M: Male. MDS: Multidimensional scaling.

To quality check for sex-specificity of our dataset, we assessed the expression of sex-chromosome linked genes, i.e. female-specific *Xist* and male-specific *Ddx3y* (Fig. 4a,b). Through sample-wise single-cell expression analysis and subpopulation-wise pseudo-bulk analysis of *Xist* and *Ddx3y* expression, we confirmed the expression of sex-specific genes in their respective samples (Fig. 4a,b). Additionally, our sex-wise comparisons confirmed that all six neutrophil subpopulations (G2-4 and G5a-c) were represented across the female and male samples in similar proportions (Fig. 4c).

### Pseudo-bulk-level analysis of sex-dimorphic neutrophil transcriptomes

More generally, to determine whether the transcriptomes of neutrophils and the underlying neutrophil subpopulations displayed sex-dimorphic expression patterns, we further analyzed the pseudo-bulk expression data. Using Multi Dimensional Scaling (MDS) as a dimensional reduction approach, we observed clear distinction between female and male samples at the global level (Fig. 4d). Further, when we compared the transcriptomes of each neutrophil subpopulation, we found similar clustering of female *vs*. male samples in all subpopulations, except for G5b (Fig. 4e and Supplementary Figure 1b), consistent with the notion that sex-dimorphic gene regulation occurs across neutrophil subpopulations. This observation suggests that the majority of neutrophil subpopulations present with sex-dimorphic transcriptional landscapes.

Together, through the technical validations described above, we show that our single-cell RNA-seq dataset of bone marrow neutrophils from adult female and male mice is of high-quality. Additionally, we observed distinct neutrophil transcriptional landscapes between females and males. This dataset will serve as a valuable resource to interrogate sex differences in neutrophil landscapes at the single-cell level and to investigate the regulatory mechanisms underlying sex-dimorphism in neutrophil biology.

## Usage Notes

Here, we generated a single-cell RNA-seq dataset of murine primary bone marrow neutrophils from adult female and male animals. From our neutrophil subpopulation marker gene expression analysis, we observed comparable expression levels of the marker genes among female and male samples (Supplementary Figure 1a). Thus, cell identities of neutrophil subpopulations seem to be preserved between the two sexes. On the other hand, our pseudo-bulk-level analysis revealed distinct transcriptional landscapes of neutrophils between female and male subjects (Fig. 4d), which is consistent with previous studies that showed sex-dimorphic transcriptomes of neutrophils via bulk RNA-seq analyses^7,8^. In addition, we also provide evidence that sex-dimorphic gene expression also occurs in different neutrophil subpopulations (Fig. 4e and Supplementary Figure 1b), and is likely to not stem solely from sex-dimorphism in underlying patterns of heterogeneity. A potential limitation of this dataset includes the relatively small sample size (n = 2 per sex), which only allows for the discovery of large sex differences. Thus, future studies with larger numbers of animals will be needed for the detection of more subtle sex-dimorphic effects. Overall, the dataset described here will be an invaluable resource to start assessing the sex differences in the gene expression profiles across neutrophil subpopulations. This dataset may also be leveraged to identify differences in RNA velocity and/or transcription factor regulon activity as a function of sex and stage of neutrophil maturation (for example, using SCENIC^48^). Importantly, this dataset represents data from sexually mature C56BL/6J animals in good health and should be treated as such when inferring potential biological responses as a function of genetic background, in response to infection or with respect to animal organismal age. Future work including profiling of neutrophils from other mouse strains (both inbred and outbred), other ages and/or health states will be important to determine the conservation of sex-dimorphic transcriptional patterns of neutrophils across biological contexts.

## Supplementary information

Supplementary Figure 1

## Data Availability

All analytical code used for processing and technical validation is available on the Benayoun Laboratory GitHub repository (https://github.com/BenayounLaboratory/Neutrophil_scRNAseq_2022). The provided R code was run and tested using R 4.1.2^27^.
