# Supplementary material for: Single-cell RNA-seq of primary bone marrow neutrophils from female and male adult mice
Source: Sci Data. 2022 Jul 23;9:442. doi: 10.1038/s41597-022-01544-7 (PMC9308797; doi:10.1038/s41597-022-01544-7)
Supplement: Supplementary file 1 — Supplementary Figure 1 [file 41597_2022_1544_MOESM1_ESM.pdf]

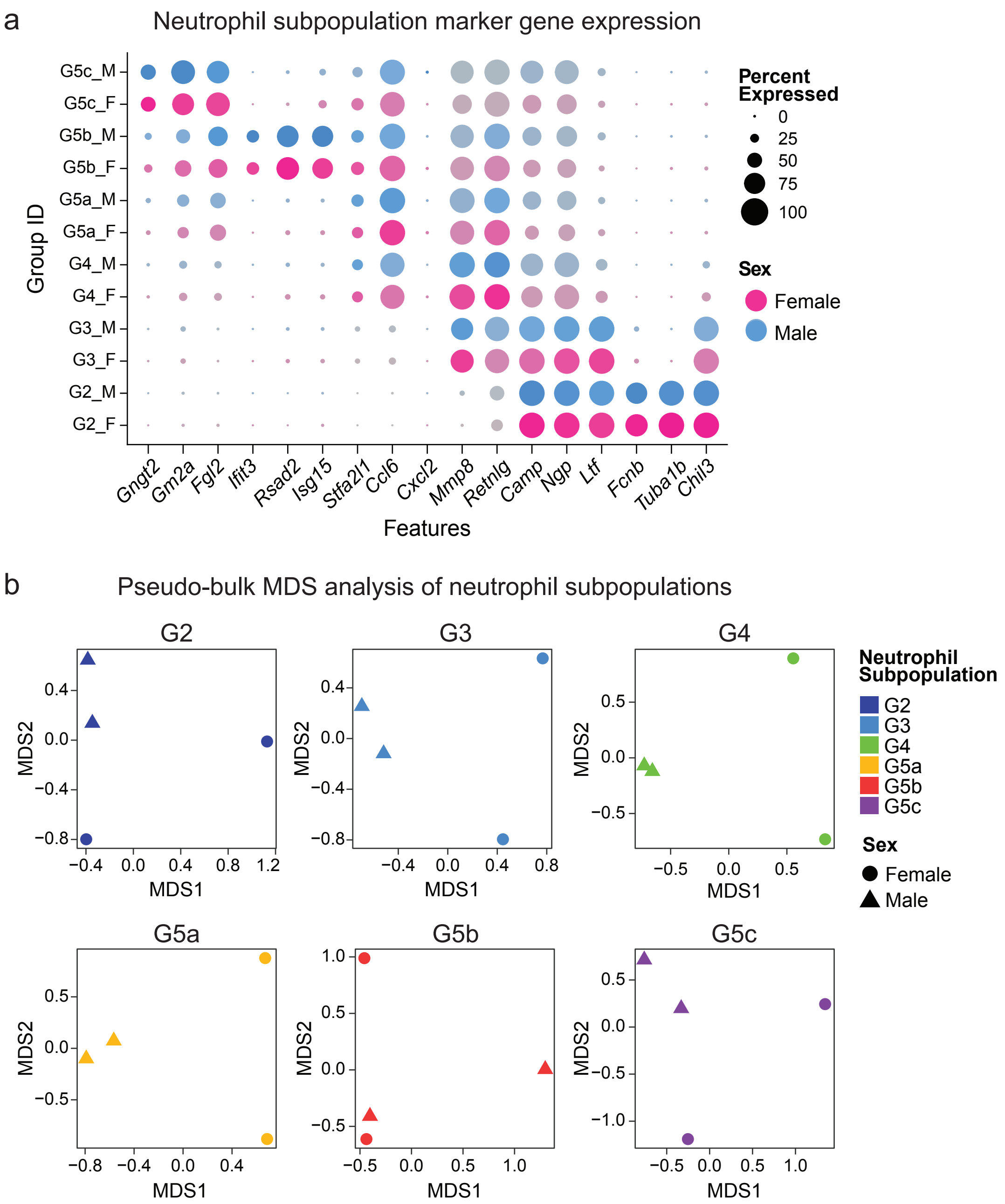

Supplementary Figure 1. Neutrophil subpopulation marker gene expression and pseudo-bulk gene expression analyses of female and male neutrophils. (a) Dot plot of scaled expression levels of neutrophil subpopulation marker genes derived from the Xie et al. dataset[34] comparing female and male samples. (b) Individual MDS plots of pseudo-bulk-level gene expression analysis comparing female vs. male transcriptomes of each neutrophil subpopulation. F: Female. M: Male.
